# Cryptic Diversity Hidden within the Leafminer Genus *Liriomyza* (Diptera: Agromyzidae)

**DOI:** 10.3390/genes9110554

**Published:** 2018-11-15

**Authors:** Antonio Carapelli, Abir Soltani, Chiara Leo, Matteo Vitale, Moez Amri, Jouda Mediouni-Ben Jemâa

**Affiliations:** 1 Department of Life Sciences, University of Siena, Via A. Moro 2, 53100 Siena, Italy; leo6@student.unisi.it (C.L.); matteo.vitale@student.unisi.it (M.V.); 2 Faculté des Sciences de Bizerte, Université de Carthage, 7021 Zarzouna Bizerte, Tunisia; soltani.abir@live.fr; 3 Biodiversity and Crop Improvement Program (BCI), International Center for Agricultural Research in the Dry Areas (ICARDA), 10112 Rabat-institutes, Morocco; M.Amri@cgiar.org; 4 Laboratoire de Biotechnologie Appliquée à l’Agriculture, INRAT, Université de Carthage, Rue Hedi Karray, 2080 Ariana, Tunisia; j_mediouni@hotmail.fr

**Keywords:** leafminers, cryptic species, species delimitation, population genetics, molecular phylogenetics

## Abstract

Leafminer insects of the genus *Liriomyza* are small flies whose larvae feed on the internal tissue of some of the most important crop plants for the human diet. Several of these pest species are highly uniform from the morphological point of view, meaning molecular data represents the only reliable taxonomic tool useful to define cryptic boundaries. In this study, both mitochondrial and nuclear molecular markers have been applied to investigate the population genetics of some Tunisian populations of the polyphagous species *Liriomyza cicerina*, one of the most important pest of chickpea cultivars in the whole Mediterranean region. Molecular data have been collected on larvae isolated from chickpea, faba bean, and lentil leaves, and used for population genetics, phylogenetics, and species delimitation analyses. Results point toward high differentiation levels between specimens collected on the three different legume crops, which, according to the species delimitation methods, are also sufficient to define incipient species differentiation and cryptic species occurrence, apparently tied up with host choice. Genetic data have also been applied for a phylogenetic comparison among *Liriomyza* species, further confirming their decisive role in the systematic studies of the genus.

## 1. Introduction

Leafminers (Diptera: Agromyzidae) are phytophagous insect pests of great economic interest worldwide and represent one of the largest fly families. Polyphagous species feed on several plantation and horticultural crops, whose leaves are tunneled by the pest larvae. Characteristic serpentine mines damage the leaflets and reduce the yearly yield of many vegetable cultivars. Among the most important leafminer flies, those of the genus *Liriomyza* (Mik, 1894) are well-known borers of a wide variety of crops. Many of them are host-specific or mine a restricted number of related plants although, among Agromyzidae, the genus adapts to the greatest number of host plants families and has a strong propensity to colonize new hosts [1].

This fly group is believed to be of Neotropic origin and comprises more than 300 species, distributed in most temperate and tropical regions of the planet. Among them, New World taxa, such as *Liriomyza huidobrensis*, *Liriomyza sativae*, and *Liriomyza trifolii* (all polyphagous) are especially troublesome for potatoes, cucurbits, and other horticultural and green house cultivars. One-third of the species is naturally found in Europe [2], where major economic damages are attributable to *Liriomyza bryoniae*, which attacks tomatoes crops. Instead, with respect to the whole Mediterranean region, *Liriomyza cicerina* is reported to be a severe pest of host plants of the family Fabaceae. Recently, in order to contain their invasive route, some taxa (such as *L. huidobrensis*, *L. sativae*, and *L. trifolii*) have been declared quarantine species in the European and Mediterranean Plant Protection Organization (EPPO) A2 list of pests [3]. Nevertheless, some *Liriomyza* species have been dislocated from their natural environment and accidentally introduced in new geographical areas, raising major concern among crop growers. To make the whole situation worse, resistance to many conventional insecticidal treatments has already been detected [4].

In general, *Liriomyza* species are morphologically uniform and therefore, especially difficult to distinguish from the congeneric taxa. Due to their homogeneous structure, neither the shape, nor the size of tunnels, built by the larvae, can be successfully applied for the systematics of the genus. The only diagnostic character, so far applied for *Liriomyza* species identification, requires dissection of males to inspect genitalia [5]. However, this analysis may be sometimes inconclusive, i.e., in the attempt to distinguish *L. huidobrensis* and *Liriomyza langei* [6], or *L. trifolii* from *L. bryoniae* and *L. sativae* [7].

From a systematic point of view, molecular data, either nuclear and mitochondrial, have been applied to investigate the phylogenetic relationships at different taxonomic levels, within Agromyzidae. Combined sets of markers have been used to provide support for the basal dichotomic division of the family in Agromyzinae and Phytomyzinae; but also, to define the phylogenetic relationships among species [1]. However, major efforts have been dedicated to defining species boundaries and, elsewhere to identify cryptic and/or invasive species, in the lack of clear morphological distinction [8,9,10]. Nowadays, molecular data are the most reliable characters required for *Liriomyza* species sorting, either using standard and multiplex PCR, and Restriction Fragment Length Polymorphism methods [10,11,12,13,14]. Presently, there are 62 species and 2008 specimens of the genus with barcodes in the Barcode of Life Data System (BOLD); however, only some taxa, such as *Liriomyza brassicae*, *Liriomyza fricki*, *L. sativae* and *L. trifolii*, are well represented in the Barcode of Life data base (>100 sequences), whereas some other species have no public *cox1* sequence at all (e.g., *Liriomyza blechi* or *Liriomyza morio*).

*Liriomyza cicerina* (Rondani 1875) affects several pulse plantations, in most temperate and tropical regions of the World and is a notorious pest of chickpea (*Cicer arietinum* L.) on which causes serious reduction of yearly crop-production and economic losses. The host plant is damaged either by the adult fly female, that punctures the leaves with its ovipositor to feed on the exudates, or by the larvae mining the mesophyll tissue, when the eggs inserted by the female on the leaf etch. Agricultural damages on chickpea production is particularly alarming for some Mediterranean farmlands. *Cicer arietinum* is an important source of human food and the World’s third most cultivated pulse crop. In the Mediterranean region, several countries (especially those of the North-African side of the sea basin) suffer the consequences of the pest activities on cultivars, with estimated loss of seed production up to 30%, every year. *Liriomyza cicerina* is native to the Mediterranean area and was formerly a parasite of *Ononis* species that switched host to chickpea, once this legume crop was introduced from Asia to Southern Europe [2]. Despite, their good-adaptation to arid and semi-arid environments, *L. cicerina* populations density increases under irrigated conditions. The fly has a polyvoltine reproductive life-cycle which is influenced by average temperature and latitudinal gradient, emerging during springtime (April) until mid-August and overwintering as pupae. Date of emergence may be earlier in warmer climate regions, with a complete life-cycle of 20–30 days and up to three overlapping generations after the first.

Molecular knowledge on *L. cicerina* is very limited with only one sequence available in both GenBank and BOLD. However, specimens of the genus have been intercepted in different hosts of Fabaceae (i.e., chickpea, lentil and faba bean) and are usually treated as a single species [15]. Therefore, either: (1) *L. cicerina* is highly polyphagous and adapts well to a vast number of hosts; or (2) more than one species is hidden within the same taxonomic name, each one with its preferred host.

In this study, we have analyzed, to our knowledge for the first time, the population genetics of several *L. cicerina* samples, collected from localities in Tunisia, where chickpea, lentil and faba bean cultivars are present, using the popular cytochrome *c* oxidase subunit I marker (*cox1*). This survey represents the first attempt to describe the genetic variability of the species in North-African Countries, and to provide a list of sequences useful for its barcode identification. Analyzed specimens were collected on different host plants (chickpea, faba bean and lentil) to establish if host-specific genetic groups form monophyletic clusters within the phylogenetic tree of the *Liriomyza* species. Moreover, distance-based, maximum likelihood and Bayesian species delimitation methods were applied to evaluate their taxonomic status and to assess the presence of unidentified species complexes. The multispecies coalescent approaches, although relying on single-locus, and frequently lessened as inaccurate or more adapt to distinguish the population structure of a species [16], still remain a good way to provide hypotheses on inter- and intraspecific boundaries in the presence of cryptic species [17]. Therefore, a combined set of nuclear (*28S* ribosomal DNA (rDNA) encoding for the large ribosomal subunit; and *CAD*, encoding for the carbamoyl-phosphate synthetase 2) and mitochondrial (*cox1*) markers have been applied in this study for: (1) single- (i.e., *cox1*, *CAD* and *28S*, individually) and multi-locus (i.e., concatenate of the three markers) species delimitation; (2) a phylogenetic comparison among congeneric species aimed at unveiling the deeper branching of *Liriomyza* lineages. Resulted genetic screening has subsequently prompted an ongoing preliminary morphological study of specimens belonging to alternative clusters in order to evaluate the occurrence of cryptic species and their relationships with the host plants (data not shown).

## 2. Materials and Methods

### 2.1. Sampling Collection, DNA Extraction and Sequencing

**Figure 1:**
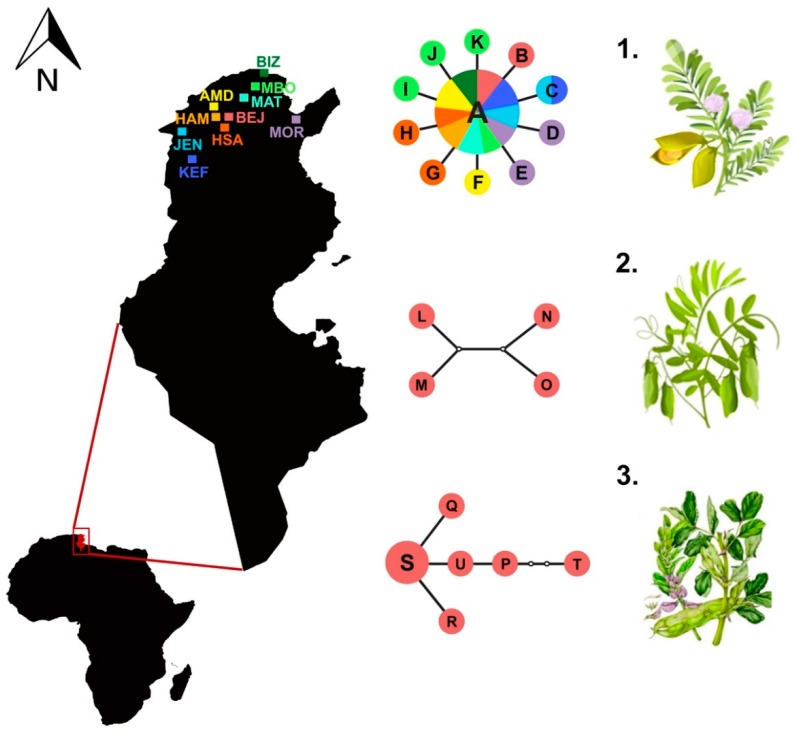
Map of Tunisia, with sampling sites represented with a three-letter code (see Table 1). In the middle, the three haplotype networks identified in the population genetics analysis of *Liriomyza cicerina*, corresponding to specimens collected on: (1) *Cicer arietinum*; (2) *Lens culinaris*; (3) *Vicia faba*, with the only exception of one specimen from lentils which share the haplotype A with those sampled on chickpea. The haplotype (circle) size is proportional to its frequency; black lines represent one nucleotide substitution, whereas white circles the missing haplotype. Plants images downloaded from [18].

Larvae of *L. cicerina* were collected, during 2016 and 2017 spring, on chickpea, faba bean and lentil from ten different localities in the North of Tunisia where these legume crops are mainly cultivated (Figure 1; Table 1). The specimens were morphologically identified by researchers from INRAT (Institut National de Recherche Agronomique de Tunis), and were preserved in alcohol for further morphological and molecular analyses. Most of samples were isolated from chickpea plants, whereas only five from lentil and eight from faba bean (Table 1).

The whole genomic DNA was extracted by means of the Wizard^®^ SV genomic DNA Purification System (Promega, Madison, WI, USA) and used to amplify the mitochondrial barcode gene *cox1*. The fragment was selected using the primers LCO (5′-GGTCAACAAATCATAAAGATATTGG-3′), and HCO (5′-TAAACTTCAGGGTGACCAAAAAATCA-3′) [19]. The total reaction volume of 25 μL contained 2.5 μL of genomic DNA, 0.5 mM of each primer, 0.2 mM of each deoxynucleotides (dNTPs, 10 mM), 2.5 mM of MgCl_2_, 5 μL of Green GoTaq Flexi buffer and 0.625 u of GoTaq Flexi DNA Polymerase (Promega). The amplifications were performed in a GeneAmp^®^ PCR System 2700 (Applied Biosystems, Foster City, CA, USA) thermal cycler. The initial denaturation step was set at 95 °C for 5 min, followed by: 35 cycles at 94 °C for 1 min, 50 °C for 1 min and 72 °C for 90 s, and a final extension step at 72 °C for 7 min.

In order to amplify both *CAD* and *28S* genes, the sequences of *Liriomyza* spp. available on Genbank were downloaded and aligned using the online tool Clustal Omega (https://www.ebi.ac.uk/Tools/msa/clustalo/) (accession numbers in Table S1 in the Supplementary Materials); primers were then identified in conserved regions. The *CAD* gene was selected with the oligo pair: Lir-CAD-53F (5′-ATGAGAAAGATGAATATGGYATGCC-3′) and Lir-CAD-689R (5′-TGRCCRCGATTACCAAATTTCAT-3′); whereas the large ribosomal subunit was amplified with: Lir-28S-31F (5′-TAGTAGTGGCGAGCGAAAAGA-3′) and Lir-28S-1366R (5′-GTATCAGTATGAGTTGCTAATTTGGG-3′). The PCRs were performed as aforementioned, varying only the elongation time for *28S* gene from 90 s to 2 min. The PCR products were then purified using the kit Wizard^®^ SV Gel and PCR Clean-up (Promega), and sequenced on both strands with a DNA Analyzer ABI 3730, at the core facility of the Biofab Research Lab (Rome, Italy). The sequences were assembled and manually corrected using Sequencher 4.4.2 (Gene Codes Corporation, Ann Arbor, MI, USA) and deposited in GenBank under accession numbers: MH878802-MH878822 for *cox1*, MH878823-MH878828 for *CAD* and MH878829-MH878830 for *28S* rDNA.

### 2.2. Assembling the Data Sets

About ten specimens from each of the ten sampling sites of this study were included in the single-locus (*cox1*) analyses, corresponding to 115 sequences, collected on *C. arietinum*, *Lens culinaris* and *Vicia faba*. Because of the uniform morphology typical of the agromyzid species, a similarity search against both the Nucleotide Basic Local Alignment Search Tool (BLASTn) and BOLD databases was performed. The initial *cox1* data set was then assembled and aligned using the online tool Clustal Omega. The alignment was manually corrected using the software Mesquite 3.51 [20] and it resulted in a 658 bp matrix, with no indels.

Moreover, about 20 specimens from Béja region were screened also for the two nuclear markers chosen for our analyses. Six different haplotypes of *CAD* gene were identified among *L. cicerina* specimens sampled on *C. arietinum*, *L. culinaris* and *V. faba* (i.e., two for each of the pest-host complex analyzed); whereas, only two different *28S* rDNA variants were obtained: one corresponding to specimens sampled on both chickpea and lentil, the other specific for specimens isolated from faba bean.

Thereafter, three single-locus and one multi-locus data sets were assembled. The *cox1* sequences obtained in the present study were aligned with those of 24 species belonging to the genus *Liriomyza* and to three agromyzid species used as outgroups: *Calycomyza majuscula*, *Calycomyza malvae* and *Cerodontha fasciata* (GenBank accession numbers and BOLD codes in Table S2 in the Supplementary Materials). The *CAD* and *28S* sequences of *L. cicerina* were individually aligned with those obtained by Scheffer et al. [1] resulting into two matrices of 591 bp and 1011 bp in length, respectively. These latter data sets were restricted only to the *Liriomyza* spp. for which the two nuclear markers were available on Genbank, and to the same outgroup taxa as before (see Table S1 in the Supplementary Materials). Finally, to further investigate the evolutionary relationships among *L. cicerina* specimens and to provide a phylogenetic reconstruction among the species of the genus, a concatenate of *cox1*, *CAD* and *28S* genes (i.e., multi-locus data set) was assembled.

In order to avoid artefacts during the phylogenetic analyses, the *28S* rDNA alignment was deprived of the hyper-variable regions using the online tool Gblocks server [21]. The 87% of the initial *28S* rDNA data set was conserved and concatenated to the *cox1* and *CAD* alignments through the software Mesquite 3.51 [20].

### 2.3. Genetic Variability

The *cox1* single-locus data set was used for the network clade analysis, performed by means of the software TCS 1.21 [22] using the default setting of 95% connection limit. The haplotype frequencies were obtained through the online tool DNA-Collapser [23]. The proportion of nucleotide changes between the haplotypes (*p*-distance) identified among *L. cicerina* specimens and against all the different outgroups was calculated using the package APE developed under the R environment [24]. The comparison between *L. cicerina* and the other species was necessary to both define the genetic variability threshold at an inter- and intra-specific level and to further avoid misidentification of the larvae during the analyses.

### 2.4. Phylogenetic Analyses

The *cox1* and *CAD* single-locus data sets, assembled as described before, were partitioned into three different charsets (1st, 2nd, and 3rd codon positions), whereas the *28S* was considered as a single partition. The software PartitionFinder 2 [25] was applied to find the evolutionary models that best fit our data sets. In order to infer a rooted topology to investigate both the putative presence of host-specific clusters within *L. cicerina* and its evolutionary relationships with the other congeneric species, the data sets were individually tested with both Maximum-likelihood (ML) and Bayesian inference (BI) methods, as implemented in RaxML 8.2.9 [26] and MrBayes 3.2 [27], respectively. The Maximum-likelihood analysis was executed using RAxML, run on the CIPRES Science Gateway V. 3.3 [28] under the GTR + Γ model with eight categories of discrete gamma distribution. The procedure included 100 independent runs of the ML analysis and 1000 replicates of the multi-parametric bootstrap. The program MrBayes 3.2 [27] was then run applying four chains for 10^6^ generations, with a sampling frequency of one tree every 1000 iterations, and with the 25% of the tree topologies discarded (burn-in step).

The multi-locus data set (i.e., a concatenate of *cox1*, *CAD* and *28S*) was further divided into seven different partitions: 1st, 2nd, 3rd codon positions for *cox1* gene; 1st, 2nd, 3rd codon positions for *CAD* gene; and, the *28S* rDNA region. The software PartitionFinder 2 [25] was again applied to identify the best evolutionary models and both ML and BI were run as described before.

### 2.5. Species Delimitation Analyses

The 109 barcode sequences, included in the *cox1* data set, were downloaded from BOLD System (Table S2 in Supplementary Materials). Given the pronounced morphological homogeneity, typical of agromyzid species, the classification of those specimens was reassessed through molecular species delimitation methods based on pairwise distances (ABGD [29], Species Identifier 1.8 [30] and TCS [22]), as well as on maximum likelihood (GMYC) and Bayesian (bPTP and BPP) optimization criteria.

The online tool Automatic Barcode Gap Discovery (ABGD, http://wwwabi.snv.jussieu.fr/public/abgd/abgdweb.html) was applied to detect the barcode gap within the distribution of pairwise distances obtained from a sequence alignment [29]. The analysis was performed using the Kimura two-parameter (K2P) model, defining the priors minimum and maximum intra-specific divergence as 0.001 and 0.05, respectively, and a gap width of 1. The species delimitation was also tested with the software Species Identifier [30], that split the sequences in clusters (i.e., putative species) based on the maximum intra-specific and the minimum inter-specific divergence identified from the barcode gene data set.

The Poisson Tree Process (PTP) was applied in order to define species boundaries based on a phylogenetic method [31]. The analyses were carried out on the web server bPTP (http://species.h-its.org/ptp/), using either the MrBayes and the RAxML topologies as input rooted tree. Each analysis was run for 500,000 MCMC generations with a burn-in value of 0.25 and performed both including and excluding the outgroup species.

The ultrametric tree, was obtained by means of the software BEAST 2.4.8 [32]. The GTR + Γ + I model was applied to each partition (1st, 2nd and 3rd codon positions) and base frequencies estimated during the analysis. A strict molecular clock was applied, defining the clock.rate based on the average mutation rate per million year identified in Brower [33], with a coalescent model of constant population size as tree prior. Two independent MCMC runs were performed, constituted of 10^6^ generations with parameters sampled every 1000 iterations and a burn-in of 25%. The two runs were combined through the BEAST package LogCombiner 2.4.8 [32]. The convergence of MCMC chains was assessed with Tracer v 1.7 [34] and the consensus tree visualized through the software FigTree v1.4.3 [35].

The ultrametric topology was then applied for the single-threshold GMYC (Generalized Mixed Yule Coalescent method [36]) analysis as well. This latter was conducted in R 3.3.2 (R Core Team 2016 [37]) with the use of the *splits* package [38].

The Bayesian Phylogenetics & Phylogeography (BPP) program was used to carry out either the single-locus (i.e., *cox1*, *CAD* and *28S* data sets, singularly analyzed) and the multi-locus (i.e., the concatenate of the three molecular markers) species delimitation analyses under the multispecies coalescent model [39]. In particular, the joint species delimitation and species-tree inference was performed (i.e., speciesdelimitation = 1, speciestree = 1; A11, [39]). The algorithm 0 and the default settings for fine-tuning parameters were used (ε = 5), as well as the species model prior 1 (i.e., uniform probability for rooted tree). Since no empirical data were available for the studied species to define appropriate priors distribution of the parameters θ (ancestral population size) and τ (root age), the species delimitation analyses were run with the following combination of gamma distributions: (1) θ: G(2: 2000), τ: G(2: 2000); (2) θ: G(1: 10), τ: G(2: 2000); (3) θ: G(2: 100), τ: G(2: 500); (4) θ: G(1: 10), τ: G(1: 10). The analyses were run for 100,000 MCMC generations, with a sample frequency of 50 and a burn-in of 1000 generations; each analysis was run twice in order to confirm the consistency of the results.

Finally, the software TCS v1.21 [22] was applied again to the *cox1* data set of those *Liriomyza* species that have been split into different clusters by the analyses, as a further evidence for the presence of putative cryptic species.

## 3. Results

### 3.1. Haplotypes and Population Genetics Analyses

A total of 115 individual sequences were obtained from specimens sampled in 10 Tunisian sites and from three different host plants (chickpea, lentil and faba bean, Table 1). Among the 21 haplotypes observed in the *cox1* data set, A is the most frequent, and shared by all populations with the exception of those specimens isolated from *V. faba* plants. In addition, most of the sampling areas shows a unique set of haplotypes, except for KEF and JEN, which share both A and C variants.

The estimated network shows three well-distinct phylogroups, corresponding to specimens collected on chickpea, lentil and faba bean, respectively (Figure 1), displaying signs of divergent selection among host plants in these leafminers. Haplotypes from specimens collected on *C. arietinum* host are arranged in a star-like network, with the haplotype A being the ancestral and most frequent variant observed, from which the haplotypes B-K differ for only one nucleotide substitution (Figure 1). Although one of the specimens isolated from *L. culinaris* has one haplotype (A) clustered with the chickpea group, all the other haplotypes identified for the lentil subset form a separated phylogroup of sequences (Figure 1) that differs for two or three nucleotide substitutions each other and for 27 to 32 nucleotide substitutions from chickpea haplotypes. Finally, the third subset is constituted only by the haplotypes detected among specimens isolated from *V. faba* host, again grouped within an exclusive haplotype sub-network (Figure 1). Within this latter cluster, the haplotype S results the ancestral and most frequent, from which all the other variants (P-R and T-U) are derived and differ for one to five substitutions (Figure 1).

Within host-specific variability of specimens collected on *V. faba* ranges from one to seven nucleotide substitutions, whereas comparisons of these latter haplotypes with those obtained from *C. arietinum* and *L. culinaris* are from 88 to 93, and from 83 to 90, respectively. The average pairwise distance values, calculated within and between the *Liriomyza* species and the three outgroup taxa (*C. majuscula*, *C. malvae* and *C. fasciata*) included in the present study, range from 0.10% (among *C. fasciata* haplotypes) to 17.53% (*Liriomyza ptarmicae* vs. *Liriomyza flaveola*) (Table 2). Within species, the average genetic distances vary from 0.10% (again *C. fasciata* haplotypes) to 1.93% (observed comparing *L. flaveola* haplotypes) (Table 2). Genetic variability among the two cryptospecies *L. huidobrensis* and *L. langei* is 5.76%. *Liriomyza strigata* and the closely related *L. bryoniae* and *L. huidobrensis* show *p*-distance values of 6.54% and 7.30%, respectively. The comparison among specimens of *L. cicerina* sampled on chickpea and lentil shows a value of about 4.60%, whereas the average genetic distances of these latter against *L. cicerina* isolated from faba bean is 13.87% and 13.13%, respectively (Table 2). It is not possible to establish a cut-off value to delimit intra- from inter-specific distances (barcoding gap), due to overlap of distances observed within and between species comparisons (see diagram on Figure 1).

### 3.2. Phylogeny of the Genus *Liriomyza*

The evolutionary models identified with the software PartitionFinder [25] were: GTR + Γ for both 1st and 3rd codon positions and GTR + I + Γ for the 2nd positions of the *cox1* single-locus data set; GTR + Γ for 1st codon position and GTR + I + Γ for 2nd and 3rd *CAD* codon positions, as well as for the entire 28S.

**Figure 2:**
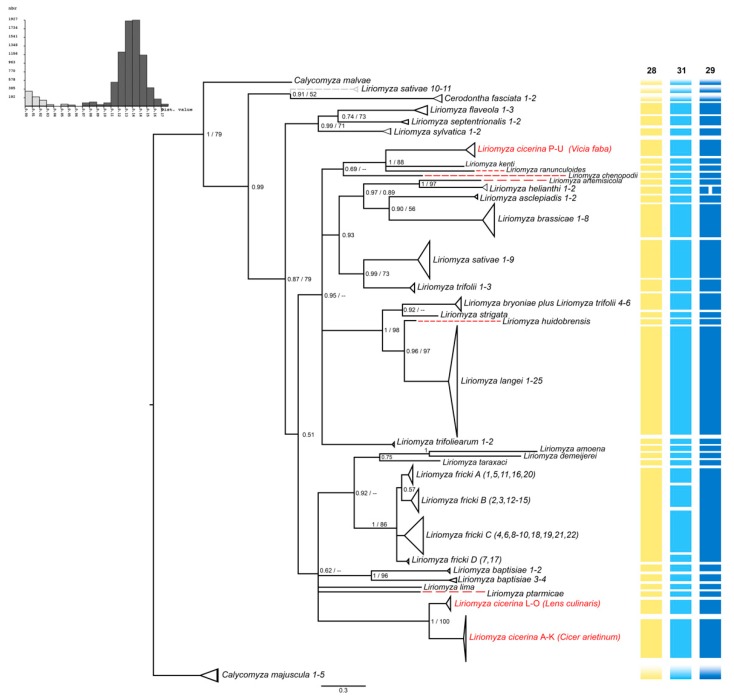
The phylogenetic tree obtained through Bayesian inference (BI), applying the cytochrome *c* oxidase subunit one (*cox1*) data set. At each node of the tree are shown the BI posterior probabilities, as well as the Maximum-Likelihood (ML) bootstrap values; dashes depict when the node is not represented in both BI and ML topologies. In red, the three *L. cicerina* clusters. Top left, the barcode gap diagram. On the right, a summary of the species delimitation methods performed on the single-locus data set. Shaded boxes indicate the outgroup species. Yellow bars show the 28 clusters detected by the programs: Automatic Barcode Gap Discovery (ABGD), Species Identifier, PTP (Poisson Tree Process) (applying BI topology and including the outgroup species) and Bayesian Phylogenetics & Phylogeography (BPP). Light blue bars display the 31 species identified by the Generalized Mixed Yule Coalescent (GMYC) model and PTP (run with BI topology and excluding the outgroup species) method. Blue bars indicate the results of PTP analyses performed with ML topology, either including and excluding the outgroup species. Top right, the total number of *Liriomyza* spp. detected by each species delimitation method applied.

**Figure 3:**
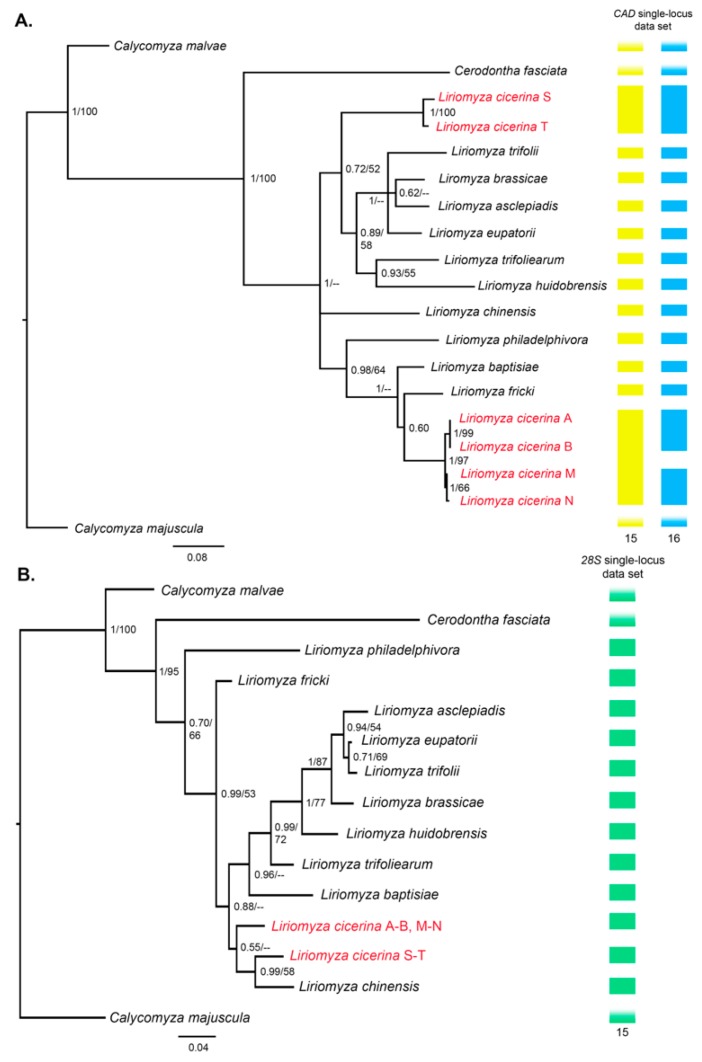
The phylogenetic trees obtained through BI, applying the single *CAD* (**A**) and *28S* (**B**) data sets. At each node of the tree, the BI posterior probabilities are shown, as well as the Maximum-likelihood (ML) bootstrap values; dashes depict when the node is not represented in both BI and ML topologies or when the bootstrap value was <50. On the right side of each tree, the results of the BPP species delimitation analyses are summarized. Yellow bars show the species clusters identified, applying the *CAD* gene, when the gamma distributions were set as: θ: G(1: 10), τ: G(1: 10) and θ: G(2: 100), τ: G(2: 500). Blue bars display the species detected with the same data set and when BPP was run with θ: G(2: 2000), τ: G(2: 2000) and θ: G(1: 10), τ: G(2: 2000). Green bars summarize the BPP outputs when the analyses were performed using the *28S* single-locus data set. Underside each series of bars, the total number of the species clusters detected is shown. Shaded boxes indicate the outgroup species.

Instead, for the multi-locus data set the following models were selected: GTR + Γ, for *cox1* 3rd positions and for *CAD* 1st and 3rd positions; GTR+I for *cox1* 2nd positions; GTR + I + Γ for the *28S* rDNA portion, the *cox1* 1st positions and for *CAD* 2nd positions. The topologies obtained with both ML and BI applied to the three single-locus data sets are fairly congruent, showing higher support for several nodes (Figure 2 and Figure 3).

When the *cox1* data set is applied to the phylogeny of the *Liriomyza* genus, most species are retrieved as monophyletic, some of them without a defined, closer, relationship with other taxa (e.g., *Liriomyza baptisiae* and *Liriomyza trifoliearum*). In both BI and ML trees, the genus *Liriomyza* results to be paraphyletic, with two haplotypes of *L. sativae* from South Korea (10 and 11: KC136117 and KC136118, respectively) clustered with the outgroup *C. fasciata* (Figure 2, posterior probability (PP) = 0.91). *L. sativae* (10-11) and *C. fasciata* (1-2) attract reciprocally at basal position of all ingroups. Removal of *C. fasciata* from the phylogenetic analysis (MrBayes and Beast analyses) does not result to monophyetic *L. sativae* (data not shown). We focus our description on the most important and notorious leafminer species, usually considered also for quarantine legislation in various countries: *L. bryoniae*, *L. huidobrensis*, *L. sativae* and *L. trifolii*. Although most of the *Liriomyza* species included in this study turn out to be monophyletic, some haplotypes of *L. trifolii* from South Korea (KC136095, KC136096 and the identical: KC136097-KC136098), referred to as *L. trifolii* 4-6, cluster with *L. bryoniae*, instead of *L. trifolii* 1-3 (EU219614, JN570506 and KR476574, respectively) from China and US (this latter cluster, sister to *L. sativae* 1-9; Figure 2). The lineage *L. trifolii* 4-6 and *L. bryoniae*, plus *L. strigata*, is sister to the group formed by the two morpho-cryptic species *L. huidobrensis* and *L. langei*. The monophyletic lineage inclusive of the *L. brassicae* and *Liriomyza asclepiadis* specimens has a strict phylogenetic relationship with the assemblage (*Liriomyza artemiscola*, *Liriomyza helianthi*). All these latter species form with *L. sativae plus L. trifolii* a monophyletic group. Other proposed phylogenetic relationships establish for: (*Liriomyza sylvatica*, (*L. flaveola*, *Liriomyza septentrionalis*)) and (*L. frickii*, (*Liriomyza taraxaci*, (*Liriomyza amoena*, *Liriomyza demeijeri*))).

The *cox1* haplotypes detected among specimens of *L. cicerina* isolated from faba bean host (P-U) cluster with *Liriomyza kenti* and *Liriomyza ranunculoides* (Figure 2), rather than being sister group of the haplotypes (A-O) obtained from larvae grown on both chickpea and lentil. The same result is observed when the *CAD* tree is inferred with either BI and ML optimization criteria (Figure 3A). The two haplotypes (S-T) of *L. cicerina* sampled on faba bean leaves are sister group of ((*L. huidobrensis*, *L. trifoliearum*), (*L. trifolii*, *Liriomyza eupatorii*, (*L. brassicae*, *L. asclepiadis*))). Again, they cluster neither with the haplotypes detected for *L. cicerina* from *C. arietinum* (A-B) nor with those of specimens sampled on *L. culinaris* (M-N, Figure 3A). A more recent common ancestor, among *L. cicerina* samples from different host plants, is instead inferred when the *28S* single-locus is applied for the analyses. Either the BI and the ML would suggest that the shared haplotype, detected between specimens from chickpea and lentil, may be the sister group of *L. cicerina* from faba bean and of *Liriomyza chinensis* (Figure 3B). However, both topologies show low support to that node (bootstrap value <50; PP = 0.50).

**Figure 4:**
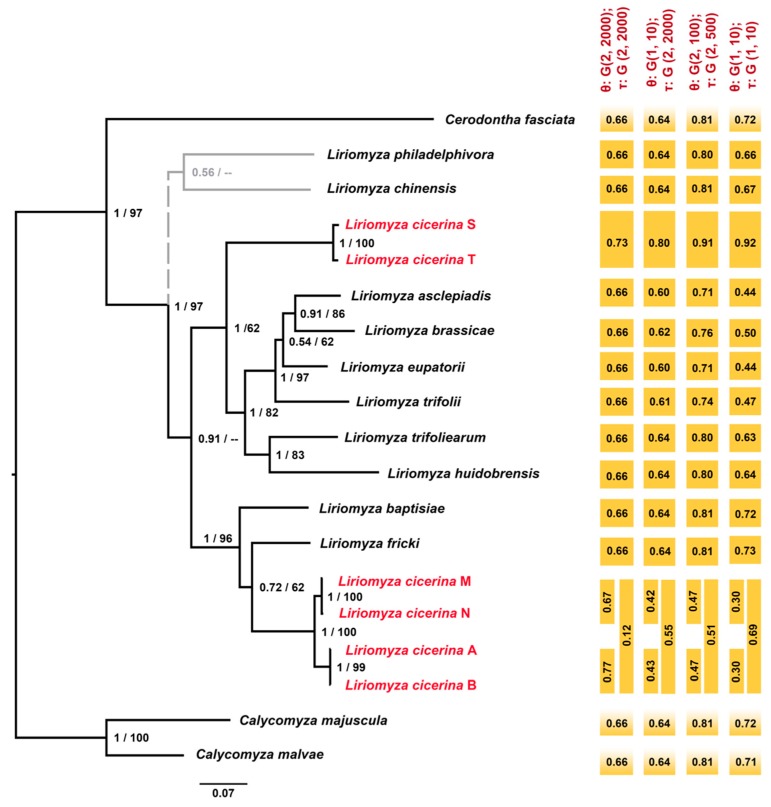
Tree topology obtained through BI, applying the concatenated data set of *cox1*, *CAD* and *28S*. At each node, the posterior probabilities and the bootstrap values are shown; dashes depict when the node was absent in the ML topology. In gray, the cluster (*Liriomyza chinensis*, *Liriomyza philadelphivora*) absent in the ML tree. In red, the three phylogroups hidden within *L. cicerina*. On the right, results of the BPP multi-locus analyses are shown, together with the respective posterior probabilities for each identified species.

Deeper relationships, along the *cox1* topologies, and the shallow ones, obtained when *CAD* and *28S* data sets are applied for *Liriomyza* phylogeny, are not shown with sufficient statistical support. Conversely, the phylogenetic trees (BI and ML), obtained using the concatenated data set of *cox1*, *CAD* and *28S* rDNA genes, show highly supported nodes for most of the branches with the genus *Liriomyza* being monophyletic (for convenience, along the text we identify the *L. cicerina* specimens with the haplotype name, previously used for the population genetics study). In addition, both analyses confirmed the host-specific split of *L. cicerina* haplotypes into two different clades. Faba bean specimens (S-T) are the sister group of a sub-clade that includes: (((*L. asclepiadis*, *L. brassicae*), *L. eupatorii*), *L. trifolii*), (*L. huidobrensis*, *L. trifoliearum*) (Figure 4). Instead, specimens isolated from both chickpea and lentil (A, B, M, N) cluster together with *L. fricki* and *L. baptisiae* being their basal group (Figure 4). Applying the concatenated data set, the two topologies, inferred with the two different optimization criteria (BI and ML), are coherent and generally fully supported. The only exception is the position of *L. chinensis* and *Liriomyza philadelphivora* (shown in gray in Figure 4) which, in turn, are sister group and basal to all the other *Liriomyza* spp. included in the present analysis (BI tree) (Figure 4), or (according to the ML topology) are independent basal lineages of the two major branches of the tree clustering with the upper branch (*L. chinensis*), or with the lower one (*L. philadelphivora*) on Figure 4.

### 3.3. Molecular Species Delimitation

The *cox1* data set analyzed in the present study was assembled with the sequences of 24 *Liriomyza* species downloaded from public data bases, and those of *L. cicerina* (obtained in our lab), *plus* three outgroups (Table S2 in the Supplementary Materials). Applied methods of species delimitation, using the mitochondrial single-locus data set, have resulted in the identification of a larger number (from 28 to 31) of putative *Liriomyza* species (Figure 2). Performed molecular analyses detect some probable misidentification of taxa. In this respect, as already observed in the phylogenetic tree, three *L. trifolii* haplotypes (identified with numbers 4-6) are joined with *L. bryoniae* within a single taxon (all methods, Figure 2); whereas others (1-3) are sister group to *L. sativae*. Given the apparent difficulties in sorting *L. trifolii* from *L. bryoniae*, we believe the former (represented by haplotypes 4-6) was misidentified and probably belong to the latter species. Similarly, two haplotypes of *L. sativae* (numbers 10 and 11) cluster in a different position of the phylogenetic tree, with respect to the remaining conspecific taxa (that are sister to *L. trifolii*) (Figure 2) and, according to molecular species-delimitation, are apparently related to the outgroup *C. fasciata*. This would also suggest a misidentification at the genus level and therefore we believe there was another (mislabeling?) error during their taxonomic identification. Whether *L. sativae* haplotypes (10-11) belong or not to a taxon of *Liriomyza*, or to a different (*Cerodontha*?) genus, is difficult to establish. Therefore, we have excluded these two sequences from the count list of the *Liriomyza* species. The ABGD, Species Identifier, TCS, PTP “MrBayes 1” (using a Bayesian input tree and a data set inclusive of three outgroups) and BPP (with alternative parameter settings) methods all detected the same 28 taxa instead of the 25 usually accepted for taxonomy (yellow bars in Figure 2). The “extra” three are retrieved thanks to the separation of the four haplotypes of *L. baptisiae*, which were split into two putative species (1 + 2 and 3 + 4); and by *L. cicerina*, that all species delimitation methods, applied to the *cox1* marker, were concordant to separate into three clusters, according to their preferred host plant: *C. arietinum*, *L. culinaris* and *V. faba* groups (Figure 2).

Additional (up to 31) species are retrieved by a limited number of methods. The two PTP “RAxML 1-2” (with or without outgroups) analyses point for a total of 29 species, due to the distinction of haplotypes 1 and 2 of *L. helianthi* into two separate taxonomic units (light blue bars in Figure 2). Whereas, the largest number (31) of species is proposed by both PTP “MrBayes 2” (without the outgroup species) and GMYC methods, due to the split of the *L. fricki* haplotypes into four presumed lineages (blue bars in Figure 2) (although none of the independent clusters is sufficiently supported by PP values).

To summarize, two species (*L. fricki* and *L. helianthi*) are frequently retrieved as single taxonomic units, but alternatively also as a complex species, depending on the applied analysis. As for *L. sativae*, a single taxon is defined only when two haplotypes (probably belonging to mislabeled specimens) are excluded from the species delimitation analyses.

Eventually, three host-specific separate lineages are currently named as *L. cicerina* according to the single-locus analyses performed using the mitochondrial barcode gene (Figure 2). Instead, when these results are tested through the BPP software, the three host-specific clusters are not fully validated. On one hand, the separation of specimens sampled on faba bean leaves from those isolated from chickpea and lentil is clearly confirmed by each analysis. On the other, the putative species, corresponding to samples from *C. arietinum* and *L. culinaris*, respectively, are validated neither by some BPP analyses running the *CAD* data set (yellow bars in Figure 3A), nor by those performed using the *28S* single-locus analyses (green bars in Figure 3B).

These latter evidences, that would treat *L. cicerina* as a species complex, are also confirmed by the multi-locus species delimitation approaches, that assign the “faba bean” specimens to an unknown *Liriomyza* species. However, it is still unclear (low PP values) whether to distinguish as separate taxonomic groups the *L. cicerina* specimens collected on chickpea and lentil (double orange bars in Figure 4).

## 4. Discussion

Despite the high interest (and economic concern) for the pest insect as source of disturbance on cultivars of Fabaceae, in the whole Mediterranean region, studies on the population genetics of *L. cicerina* are essentially missing. Traditional view based on the fly interaction with its main affected host, would suggest a close relationships of *L. cicerina* with *C. arietinum*. However, at least one record of adult stages of this leafminer species emerging from *V. faba* has been observed in Egypt at the beginning of this century [15]; and other plants of the family Fabaceae, related to each other, are also considered hosts for the fly larvae. Levels of genetic differentiation among the *L. cicerina* specimens, affecting alternative host plants, are still unknown, and no systematic study has been performed to evaluate whether only one polyphagous taxon mines several plants or if more than one species is present, each one with its preferred host to feed on. On the other hand, previous studies on other congeneric taxa also imply that distinct cryptic species may be hidden within the same species-complex [40] and recommend a systematic revision to delimit dubious inter and intra-specific relationships [41]. Furthermore, within-species differentiation of *Liriomyza* spp., also relate to the host choice, e.g., at least one record of a *L. trifolii* population, apparently specialized to feed only on pepper, led to the speculation that new host affiliation may emerge from polyphagous lineages and be relevant to arise incipient (cryptic) species differentiation [40]. In addition, the genus *Liriomyza* is considered the Agromyzidae lineage with the largest number of host-plant families utilized and with marked tendency towards polyphagy [10].

In this study, molecular evidence attempts to define the systematic relationships among three different groups of host-specific larvae identified as *L. cicerina*.

Molecular analyses are particularly useful for the species identification of the genus, due to the uniformity of the morphological characters, and also because collected data can be obtained from all life stages and do not necessarily rely on the study of the reproductive apparatus of the male adults (the only diagnostic character applied with some effectiveness for species sorting). Genetic divergence observed among (*cox1*) haplotypes in the Tunisian population of the species collected on *V. faba* with respect to all other congeneric samples (including those mining the leaves of chickpea and lentil) spans from ~11% to 17%. These values are largely in the range of between-species comparisons (Table 2), with the highest similarity detected with respect to *L. kenti* and *L. ranunculoides*. Furthermore, nucleotide divergence between *L. cicerina* whose larvae were sampled on *C. arietinum* and *L. culinaris* is around 5%; a very similar value to that (6%) detected among the two morphocryptic and morphologically indistinguishable species *L. huidobrensis* and *L. langei* [10], or to the 7–8% observed among the closely related *L. bryoniae*, *L. huidobrensis*/*L. langei* and *L. strigata*. Therefore, host-associated divergence in mitochondrial sequences has been detected (Figure 1), with the only exception of one haplotype belonging to the “chickpea” network cluster, that is also present in a specimen collected on lentil.

The application of the species delimitation methods, using several molecular data, returns a substantially concordant pattern of species diversity, with respect to the traditional systematics of the group. Most of the species are recovered as separate taxonomic units, with few exceptions. Among them, one method (PTP) supports the subdivision of *L. helianthi* into two species (although with the lack of statistical support). Two methods (PTP and GMYC) propose as many molecular species as the four phylogroups of *L. fricki* obtained on Figure 2. As in the case of *L. cicerina*, all the species delimitation approaches have split the four *L. baptisiae* haplotypes into two different groups (1 + 2 and 3 + 4), generally with a high statistical support (Figure 2). These results are also supported by the genetic distance comparison among the two clusters (12.77%, Table 2), thus suggesting the presence of cryptic species so far identified as *L. baptisiae*. Apart from the above cases and the misidentification/mislabeling problems, probably affecting the position over the phylogenetic tree of some *L. sativae* and *L. trifolii* samples, both single- and multi-locus analyses are relatively concordant each other and lined up with traditional taxonomic subdivision of species. Phylogenetic relationships confirm the sister group *L. huidobrensis*/*L. langei* with *L. bryoniae*/*L. strigata*, and beween *L. sativae* and *L. trifolii*, as also proposed on morphological ground, i.e., the similarity of the male genitalia (especially in the shape of the distiphallus), the body color and the fine structure of the larvae’s spiracles (e.g., see Groups 1 and 2 larvae in International Plant Protection Collection, 2016 [42]).

According to the genetic distances parameters, phylogenetic studies and most of the species delimitation methods herein applied, the specimens identified as *L. cicerina* from *V. faba* host are probably belonging to a different *Liriomyza* species that clusters together with *L. kenti* and *L. ranunculoides* (not with the other two *L. cicerina* phylogroups). However, it is not possible to establish with certainty if the specimens collected on faba bean belong to a new species of the genus; indeed, not all the species, so far described with a morphological approach, are further characterized from a molecular point of view. However, the distinction between them and the other specimens of the *L. cicerina* complex is fully supported by all the species delimitations approaches (i.e., either the discovery and the validation tools). The same applies for the three single-locus data sets, tested either individually and concatenated for the species delimitation, as well as for the gene tree inference (Figure 2 and Figure 3). In this respect, only a more in-depth morphological analysis may be conclusive to establish if a new *Liriomyza* species should be described or if the specimens collected from *V. faba* can be assigned to an already-known taxon, so far never analyzed for molecular taxonomy and phylogeny.

Instead, the taxonomic status of the specimens collected on *C. arietinum* and *L. culinaris* is still questionable. Approaches based on different data, assumptions and algorithms are not unanimous in defining these lineages as well differentiated groups. Therefore, it is likely that incipient species may have been collected on lentil and chickpea, respectively. These two are reciprocally sister group in all the phylogenetic trees, obtained either with single- and multi-locus data sets, as well as applying both BI and ML (Figure 2, Figure 3 and Figure 4). Moreover, one specimen collected on lentil, shows the most frequent haplotype (A), thus clustering within the sub-network of the “chickpea” phylogroup (Table 1, Figure 1). However, either the genetic distances and most of the species delimitation methods would suggest the presence of two different species (Table 2, Figure 2 and Figure 3A); other approaches would instead confirm the occurrence of a single *L. cicerina* taxon (Figure 3 and Figure 4).

According to these conflicting results and following the unified species concept [17], it is possible to speculate that: (1) incipient species diversification may be occurring, but still with a certain degree of gene flow still active; and/or (2) the most frequent haplotype A, being the only one found on both chickpea and lentil, belong to a polyphagous and less host-restricted genetic group.

It is worth to notice, that all samples processed in this study have been collected on cultivars and therefore, it is difficult to state what is the impact of the agricultural activities on the genetic structure and pest mobility of the species. Haplotypes network analysis points for the existence of three defined sub-clades, with several singletons that appear to be associated to restricted geographic areas.

Molecular clock estimates, applying the generalized insect mitochondrial DNA estimate of 1.5–2.3% sequence divergence rates per Myr (Million years) [33], date the separation of *L. cicerina* collected on faba bean from *L. kenti* and *L. ranunculoides* between 4.9 to 7.6 Myr ago. Whereas the separation of “chickpea” and “lentil” haplotypes occurred 1.9 to 3.1 Myr ago. These latter estimates conform with those observed among the well-known cryptic species *L. huidobrensis* and *L. langei*, that diverged around 2 Myr ago [41]. In conclusion, proposed species delimitation and population genetics studies highlight that at least two cryptic species are hidden under the taxonomic name *L. cicerina*, which is actually a species complex composed of closely related taxa, very similar in appearance to the point that the boundaries between them are often unclear and presently only distinguishable on molecular ground.

As a matter of fact, a preliminary morphological analysis applied to both insect pests attacking lentil and chickpea crops, suggests that the male distiphallus is with distal lobes meeting their rims to their bases, as expected in *L. cicerina* [2,43]; whereas that of the specimens collected on faba bean is paired and sac-shaped (as seen from the ventral side). In addition, larval stages of the specimens collected on faba bean show morphological characters (i.e., the pigmentation) that are not conclusive for taxonomic diagnosis (unpublished data).

It is worth to notice that *L. huidobrensis* is one of the most polyphagous species of the genus, with several records of pest activity on faba bean [6]. In this respect, we have initially tested what was the degree of genetic affinity between this species and the *Liriomyza* specimens collected on faba bean in Béja. Eventually, phylogenetic and genetic distance analyses both suggest that the *Liriomyza* samples (haplotypes P-U; Table 2, Figure 2, Figure 3 and Figure 4) collected on faba bean do not correspond to *L. huidobrensis*, for four main reasons: (1) the placement within the phylogenetic trees (both single- and multi-locus analyses) in an independent position with respect to the other *L. huidobrensis* downloaded from public databases; (2) this latter species, conventionally clusters with *L. langei* commonly considered its sibling species; (3) the genetic distances between the Tunisian *Liriomyza* specimens collected on faba bean and *L. huidobrensis* (12–13%) are within the range of interspecific comparisons; (4) none of the species delimitation analyses clusters within a single molecular taxon *Liriomyza* “faba bean” samples and *L. huidobrensis* ones (Figure 2, Figure 3 and Figure 4).

Proposed results point towards the urge of new systematic analyses in order to evaluate if neglected morphological characters useful for species discrimination may occur. Molecular data applied for this study may be functional for barcode identification and represent a useful tool for future progress and to acquire knowledge on the evolution of such a complicated leafminer genus.

## Figures and Tables

**Table 1:** List of the Tunisian sampling sites, with respective labels and geographic coordinates; Béja, Amdoun, Hamrounia and Hamem Sayela are sites of the Béja Region; for each area the host plant, the number of specimens analyzed are listed, as well as the haplotypes identified in this study.

Collection site	Label	Coordinates	Host Plant	n	cox1 Haplotypes
Béja	BEJ	36°43′ N; 9°10′ E	*Cicer arietinum*	10	A(9), B(1)
			*Lens culinaris*	5	A(1), L(1), M(1), N(1), O(1)
			*Vicia faba*	8	P(1), Q(1), R(1), S(3), T(1), U(1)
Amdoun	AMD	36°46′ N; 9°05′ E	*Cicer arietinum*	13	A(12), F(1)
Hamrounia	HAM	36°72′ N; 9°11′ E	*Cicer arietinum*	10	A(10)
Hamem Sayela	HSA	36°39′ N; 9°8′ E	*Cicer arietinum*	10	A(8), G(1), H(1)
Le Kef	KEF	36°11′ N; 8°42′ E	*Cicer arietinum*	12	A(8), C(1)
Mornag	MOR	36°41′ N; 10°1′ E	*Cicer arietinum*	10	A(8), D(1), E(1)
Oued Meliz	JEN	36°28′ N; 8°33′ E	*Cicer arietinum*	10	A(9), C(1)
Mateur	MAT	37°02′ N; 9°39′ E	*Cicer arietinum*	10	A(10)
Bizerte	BIZ	37°16′ N; 9°52′ E	*Cicer arietinum*	10	A(10)
Menzel Bourguiba	MBO	37°9′ N; 9°47′ E	*Cicer arietinum*	10	A(7), I(1), J(1), K(1)

**Table 2:**
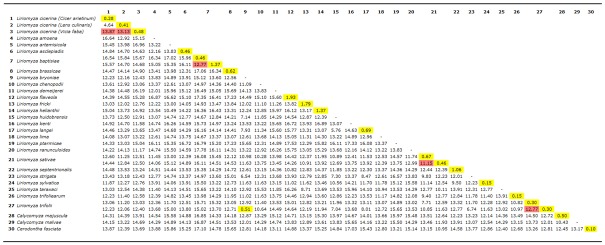
Percentage of average pairwise distance matrix; in yellow are highlighted values of intra-specific divergence; in red unexpected values of inter-specific divergence within the same species complex.

## Supplementary Materials

The following are available online at http://www.mdpi.com/2073-4425/9/11/554/s1, Table S1: List of *Liriomyza* and outgroup species included in the single-(*CAD* and *28S*) and multi-locus analyses and applied to design primer pairs to amplify and sequence nuclear markers, Table S2: List of the 24 *Liriomyza* and outgroup species and haplotype labels, included in the *cox1* single-locus analyses.
